# Arterial Spin Labeling Imaging for the Parotid Glands of Patients with Sjögren’s Syndrome

**DOI:** 10.1371/journal.pone.0150680

**Published:** 2016-03-09

**Authors:** Yukiko N. Kami, Misa Sumi, Yukinori Takagi, Miho Sasaki, Masataka Uetani, Takashi Nakamura

**Affiliations:** 1 Department of Radiology and Cancer Biology, Nagasaki University School of Dentistry, Nagasaki, Japan; 2 Department of Radiology, Nagasaki University Graduate School of Biosciences, Nagasaki, Japan; University of Kentucky, UNITED STATES

## Abstract

Sjögren’s syndrome (SS) is characterized by hypofunction of the salivary and lacrimal glands. The salivary function is largely dependent upon the blood supply in the glands. However, the diseased states of the gland perfusion are not well understood. The arterial spin labeling (ASL) technique allows noninvasive quantitative assessment of tissue perfusion without the need for contrast agent. Here, we prospectively compared the perfusion properties of the parotid glands between patients with SS and those with healthy glands using ASL MR imaging. We analyzed salivary blood flow (SBF) kinetics of 22 healthy parotid glands from 11 volunteers and 28 parotid glands from 14 SS patients using 3T pseudo-continuous ASL imaging. SBF was determined in resting state (base SBF) and at 3 sequential segments after gustatory stimulation. SBF kinetic profiles were characterized by base SBF level, increment ratio at the SBF peak, and the differences in segments where the peak appeared (SBF types). Base SBFs of the SS glands were significantly higher than those of healthy glands (59.2 ± 22.8 vs. 46.3 ± 9.0 mL/min/100 g, p = 0.01). SBF kinetic profiles of the SS glands also exhibited significantly later SBF peaks (p < 0.001) and higher SBF increment ratios (74 ± 49% vs. 47 ± 39%, p = 0.04) than the healthy glands. The best SBF criterion (= 51.2 mL/min/100 mg) differentiated between control subjects and SS patients with 71% sensitivity and 82% specificity. Taken together, these results showed that the SS parotid glands were mostly hyperemic and the SS gland responses to gustatory stimulation were stronger and more prolonged than those of the healthy glands. The ASL may be a promising technique for assessing the diseased salivary gland vascularization of SS patients.

## Introduction

Sjögren’s syndrome (SS) is a multisystem autoimmune disorder symptomatically and characterized by hypofunction of the salivary and lacrimal glands [1]. The salivary glands receive an innervation from cholinergic parasympathetic and sympathetic nerves to evoke the secretion of saliva by acinar cells and the release of stored proteins from acinar and duct cells. On the other hand, the fluid component of saliva is supplied by a dense network of blood vessels, which also receive innervation from parasympathetic and sympathetic nerves. Therefore, it can be expected that the blood supply (perfusion) of the salivary glands is disordered in patients with SS. However, the diseased states of the gland perfusion in patients with SS are not well understood.

Ultrasonography (US) has been used for assessing the perfusion properties of the salivary glands of patients with SS [2, 3]. Unfortunately, these studies did not provide sufficient merit for diagnosing SS. However, Jousse-Joulin et al reported the usefulness of US in the follow up after therapy [4, 5].

The arterial spin labeling (ASL) technique allows noninvasive quantitative assessment of tissue perfusion without the need for contrast agent administration [6, 7]. In ASL, the protons in blood are magnetically labeled, and are used as intrinsic tracers to measure the tissue blood flow volume. Histologic features of salivary glands of patients with SS are defined as a focal lymphocyte aggregation associated with chronic inflammation and acinar cell destruction in the gland parenchyma, which may cause alterations of vascular physiology of the involved glands, such as dilatation of the gland vessels and increases in blood flow volume. Furthermore, vascular permeability may also be altered in the SS glands, affecting the diffusion of low molecular weight contrast agent and the estimation of intravascular and extravascular blood volumes. The major advantages of ASL are that the technique can directly determine the intravascular blood volume (red blood cells) not affected by vessel permeability, and can also allow repeated acquisitions in the same patient because intravenous contrast agents are not necessary.

The application of ASL technique in the assessment of salivary gland perfusion has been very limited. Schwenzer et al [8] used this technique for the measurement of blood flow rates in healthy parotid glands. However, the results are debatable because the parotid blood flow rates obtained by the study were much higher than those of the brain.

Here, we applied the pseudo-continuous ASL (pCASL) technique to assess the salivary blood flow (SBF) kinetics of the parotid glands in patients with SS compared with that of healthy parotid glands.

## Materials and Methods

### Subjects

This prospective study was approved by the ethics committee of Nagasaki University. All patients gave their signed written informed consent to participate in the study, as approved by the ethics committee. We selected 16 patients from those who presented with sicca symptoms (dry mouth/dry eyes) between April 2012 and August 2013, were diagnosed with SS according to the American-European Consensus Group (AECG) criteria (n = 14) or based on the Japanese criteria (n = 2) [1, 9], and agreed to participate in the study and provided written informed consent. We also enrolled 13 volunteer subjects, who agreed to participate in the study and provided written informed consent. We also confirmed that the parotid glands of the controls were normal on T1- and fat-suppressed T2-weighted MR images that were performed along with the ASL MR imaging. We did not match the ages of the Control and SS patient groups (Table 1). Of these, 2 patients with SS and 2 healthy subjects were excluded from this study because of poor ASL image quality due to motion artifacts. Consequently, we analyzed ASL images of 28 parotid glands from 14 patients with SS (14 women; average age, 63 ± 11 years), and 22 healthy glands from 11 volunteers (11 women; average age, 53 ± 6 years).

**Table 1:** Clinical profiles of 11 volunteers and 14 SS patients.

	age	Saxon test (g/2 min)	dry mouth symptom	dry eye symptom	Schirmer test	Ocular staining	SS-A	SS-B	Lip biopsy	MR grade (L/R)	SS criteria	Comorbid disease
Volunteers												
1	50	2.87	-	-	ne	ne	ne	ne	ne	G0/G0	-	-
2	50	7.38	-	-	ne	ne	ne	ne	ne	G0/G0	-	-
3	60	3.79	-	-	ne	ne	ne	ne	ne	G0/G0	-	-
4	58	8.35	-	-	ne	ne	ne	ne	ne	G0/G0	-	-
5	53	7.38	-	-	ne	ne	ne	ne	ne	G0/G0	-	-
6	51	4.81	-	-	ne	ne	ne	ne	ne	G0/G0	-	-
7	54	4.25	-	-	ne	ne	ne	ne	ne	G0/G0	-	-
8	59	5.65	-	-	ne	ne	ne	ne	ne	G0/G0	-	-
9	54	3.46	-	-	ne	ne	ne	ne	ne	G0/G0	-	-
10	55	3.23	-	-	ne	ne	ne	ne	ne	G0/G0	-	-
11	38	9.32	-	-	ne	ne	ne	ne	ne	G0/G0	-	-
	53 ± 6^a^	5.50 ± 2.15^b^										
SS patients												
12	73	0.15	+	nd	ne	ne	+	-	+	G1/G1	AE	P-SS
13	48	1.93	+	+	+	-	+	-	-	G1/G1	AE	P-SS
14	44	3.99	+	nd	+	ne	+	-	ne	G1/G1	AE	RA
15	87	1.70	+	+	+	+	-	-	+	G1/G1	AE	RA
16	61	1.80	+	nd	ne	ne	+	+	ne	G2/G2	AE	P-SS
17	62	0.36	+	+	+	+	-	-	+	G2/G2	AE	SD
18	64	1.76	+	+	ne	ne	ne	ne	ne	G1/G2	J	SD
19	63	0.96	+	-	ne	ne	+	ne	ne	G1/G2	J	P-SS
20	56	1.72	+	nd	ne	ne	+	+	ne	G3/G3	AE	P-SS
21	76	0.64	+	+	+	ne	+	-	ne	G3/G3	AE	P-SS
22	56	0.77	+	+	+	ne	+	+	ne	G3/G3	AE	P-SS
23	72	0.33	+	+	ne	ne	+	-	-	G4/G4	AE	P-SS
24	59	1.31	+	nd	+	ne	+	-	+	G4/G4	AE	P-SS
25	60	0.43	+	+	ne	ne	+	ne	ne	G4/G4	AE	P-SS
	63 ± 11^a^	1.28 ± 0.97^b^										

ne, not examined; nd, not determined; +, positive test; -, negative test; Saxon test, normal range >2 g/2 min; SS-A and SS-B, anti-SS-A/Lo and anti-SS-B/Ra autoantibodies; P-SS, primary SS; RA, rheumatoid arthritis; SD, scleroderma. In the MR grade column, the left (L) and right (R) salivary glands were separately categorized. AE and J in SS criteria column indicate the corresponding patients were diagnosed as SS based on the American-European Consensus Group (AE) or Japanese (J) criteria, respectively.

^a, b^, Significant differences in ages (p = 0.0139, t-test) and salivary flow rates (p <0.0001; t-test) between healthy subjects and SS patients.

Clinical profiles of the patients with SS were assessed by serological tests (positive anti-SS-A/Ro and/or anti-SS-B/La antibodies), labial gland biopsy (≥1 lymphocyte foci per 4 mm^2^ area of gland parenchyma), ocular staining with fluorescence dye or lissamine green (score ≥3), and MR imaging gradings of SS (Table 1). The total salivary function of the healthy subjects and SS patients was assessed with the Saxon test, and a salivary flow rate less than 2 g/2 min was considered an indicator of impaired salivary gland function. The volunteers underwent only the Saxon test. Each of the parotid glands of volunteers and patients with SS were categorized by a radiologist, as Grade 0 to Grade 4 based on the extent of fat infiltration in the glands of T1-weighted and T2-weighted MR images [10].

### ASL MR Imaging

**Fig 1:**
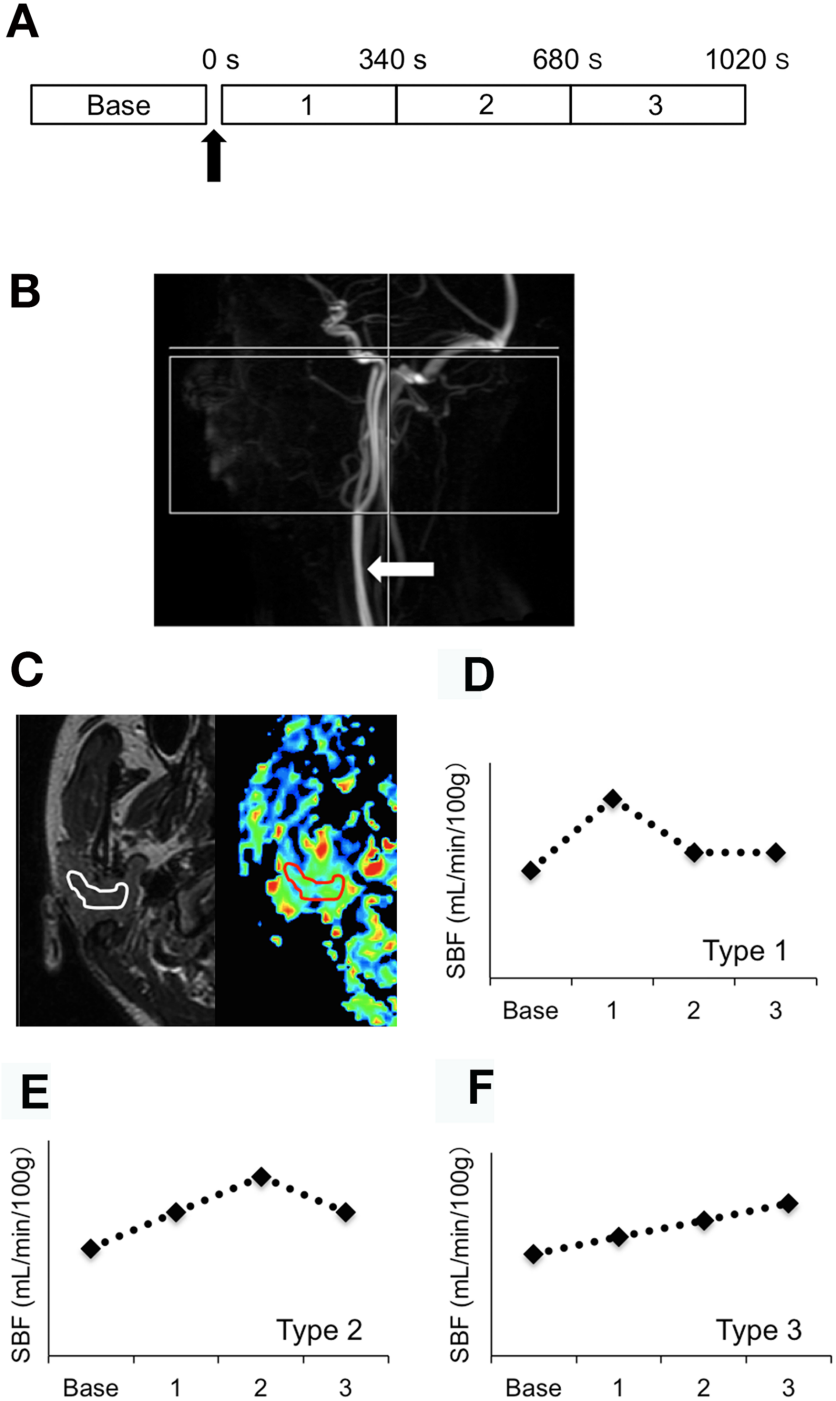
ASL MR imaging of parotid gland. A, Schema shows gustatory stimulation and ASL MR image acquisition protocol. B, Time-of-flight (TOF) MR angiogram shows common, external, and internal carotid arteries. Arrow indicates level of spin labeling (common carotid artery). Rectangular area indicates image acquisition area including parotid gland. C, Axial T2-weighted MR image (left panel) and axial color salivary blood flow (SBF) map (right panel) of healthy parotid gland. A free-hand region of interest (ROI) was drawn on the parotid gland parenchyma so that the ROI included as much of the gland area as possible but did not included the periphery of the gland, or the retromandibular vein, or the external carotid artery. The ROI placed within the parotid gland area on T2-weighted MR image is copied and pasted onto SBF map. We used 4 sequential SBF maps for the ASL analysis, including one SBF map that contained the maximum gland area; the other 3 maps were from upper and lower slices neighboring the map containing the maximum gland area. Subsequent SBF analysis was performed using the ImageJ software (http://imagej.nih.gov/). The SBF was determined by averaging the SBF from 4 ASL maps and expressed in mL/min/100 g for each of the 4 segments (base, first, second, and third). D-F, Classification of SBF kinetics profile of parotid gland. SBF peaks appear in the first (Type 1, D), second (Type 2, E), or third (Type 3, F) segment. SBF kinetics profiles are additionally characterized by base SBFs and increment ratios from base SBF to peak SBF [(peak SBF—base SBF) (Type 1, D), sec.

ASL imaging was performed on the 3.0-T MR scanner equipped with Head-Neck-Spine Array Coil (Signa EXCITE 3.0T; GE Healthcare). Perfusion assessment of the parotid gland was performed before the salivary stimulation with 100% lemon juice containing 6.5% citric acid (base segment) and in 3 subsequent sequential segments after the stimulation (0–340 s, 340–680 s, and 680–1020 s) (Fig 1A). The lemon juice was provided by placing a cotton roll containing 2.5 mL juice (163 mg citric acid) onto the upper surface of the tongue during the 3 subsequent segments of the ASL imaging.

We used the following parameters for the ASL MR imaging of the parotid glands: TR = 4589 ms, TE = 14 ms, number of excitation = 4, flip angle = 155°, acquisition matrix size = 128 × 128, FOV = 240 mm, label type = parallel slab, label gap = 20 mm, label duration = 1500 ms, and post label delay = 1525 ms. The labeling plane was set parallel to and 20 mm proximal to the imaging volume and perpendicular to the common carotid artery (Fig 1B). The scan time for the ASL imaging in each segment was 5 min and 40 s for 24 slices.

### Image Analysis

ASL perfusion data were analyzed on the Advantage Windows workstation using FuncTool version 9.4 software (GE Healthcare). The ASL application for estimating blood flow is originally supplied with a set of pre-defined protocols [equation 1 in supplementary method], which contain input parameters, including a T1t value (= 1.2 s) of the brain tissue (typical of gray matter). Therefore, the algorithm was designed specifically for the cerebral blood flow (CBF) prediction and different T1t values should be used for tissues with different T1 values. In addition, the parotid glands contain varying amounts of fat tissue, which increases with age and also with increases in severity of SS gland disease [5]. Increases in fat tissue contents in the gland decrease the T1 values of the gland parenchyma. If the pre-set T1t value is used for predicting the parotid blood flow (SBF) of healthy and SS parotid glands, the obtained SBF values would be underestimated. Accordingly, the T1 values of the parotid glands should be separately calculated for patients with different gland disease having different amounts of fat tissues (i.e., G0—G4 glands) and the values should be used for adjusting the obtained ASL data (S1 Text).

T1 relaxation time measurement was performed in 4 (8 glands) of the healthy volunteers, and 1 (2 glands) of the G1, 3 (6 glands) of the G2, 1 (2 glands) of the G3 and 1 (2 glands) of the G4 SS patients as previously described [8].

The subsequent analysis on SBF maps was performed using the ImageJ software (http://imagej.nih.gov/).

### SBF Profile Characterization

SBF profiles were qualified by analyzing the base SBF and the increment ratio from the base to the peak SBF (Fig 1D–1F). The SBF profiles were categorized into 3 types in terms of in which segment the SBF peak appeared: Type 1 glands showed the SBF peak during the first segment (0–340 s after gustatory stimulation); Type 2 glands during the second segment (340–680 s); and Type 3 glands during the third segment or later (>680 s).

### Intraobserver and Interobserver Errors

Intraobserver and interobserver errors were assessed by calculating the percent coefficient of variation (% CVs) of SBF after the same or different radiologists repeatedly placed ROIs on the gland areas. For the intraobserver error assessment, one radiologist placed an ROI onto each of the gland areas of SBF maps that were obtained from 10 left parotid glands (10 control subjects); for the interobserver error assessment, two radiologists including the one who participated in the intraobserver assessment repeatedly (3 times) placed ROIs onto each of the gland areas of the same SBF maps (n = 10). The average % CVs were calculated from the SBF data obtained from different glands (intraobserver errors) and from different observers (interobserver errors).

### Statistics

The normal distribution and equal variance of the data were evaluated by using Shapiro-Wilk test and Levene test, respectively. Differences in base SBF between healthy and SS glands, and between different grades were analyzed by using Welch test and Tukey-Kramer honest significant difference (HSD) test, respectively. Differences in distribution of SBF kinetic profiles between healthy and SS glands were analyzed by using Fisher’s exact test. Mann-Whitney U test was also used to compare increment ratios between healthy and SS glands. Receiver operating characteristic (ROC) curve analysis was performed for assessing the diagnostic performance of base SBF values in discriminating SS patients from volunteers with healthy glands, by calculating diagnostic abilities (sensitivity, specificity, accuracy, negative and positive predictive values) and areas under the curve (AUC). Correlations between SBFs and clinical features were analyzed by using Pearson’s correlation test or Spearman’s rank correlation test. JMP Pro software (version 11.2, SAS Institute) was used for all the statistical analyses.

## Results

### T1 Relaxation Time

T1 values of G3 (814.7 ms, n = 2) + G4 (588.6 ms, n = 2) SS glands (701.6 ± 144.3 ms, n = 4) were significantly lower than those of healthy glands (923.0 ± 63.0 ms, n = 8) (p = 0.022) and those of G1 (1026.9 ms, n = 2) + G2 (938.2 ms, n = 6) SS glands (960.4 ± 150.9 ms, n = 8) (p = 0.008). However, the values were similar between the healthy glands and G1 + G2 SS glands (p = 0.81).

### Intraobserver and Interobserver Errors

Intraobserver and interobserver errors in measuring SBF were 2.26 ± 1.79% and 2.93 ± 3.28%, respectively.

### Base SBFs of Healthy and SS Parotid Glands

**Fig 2:**
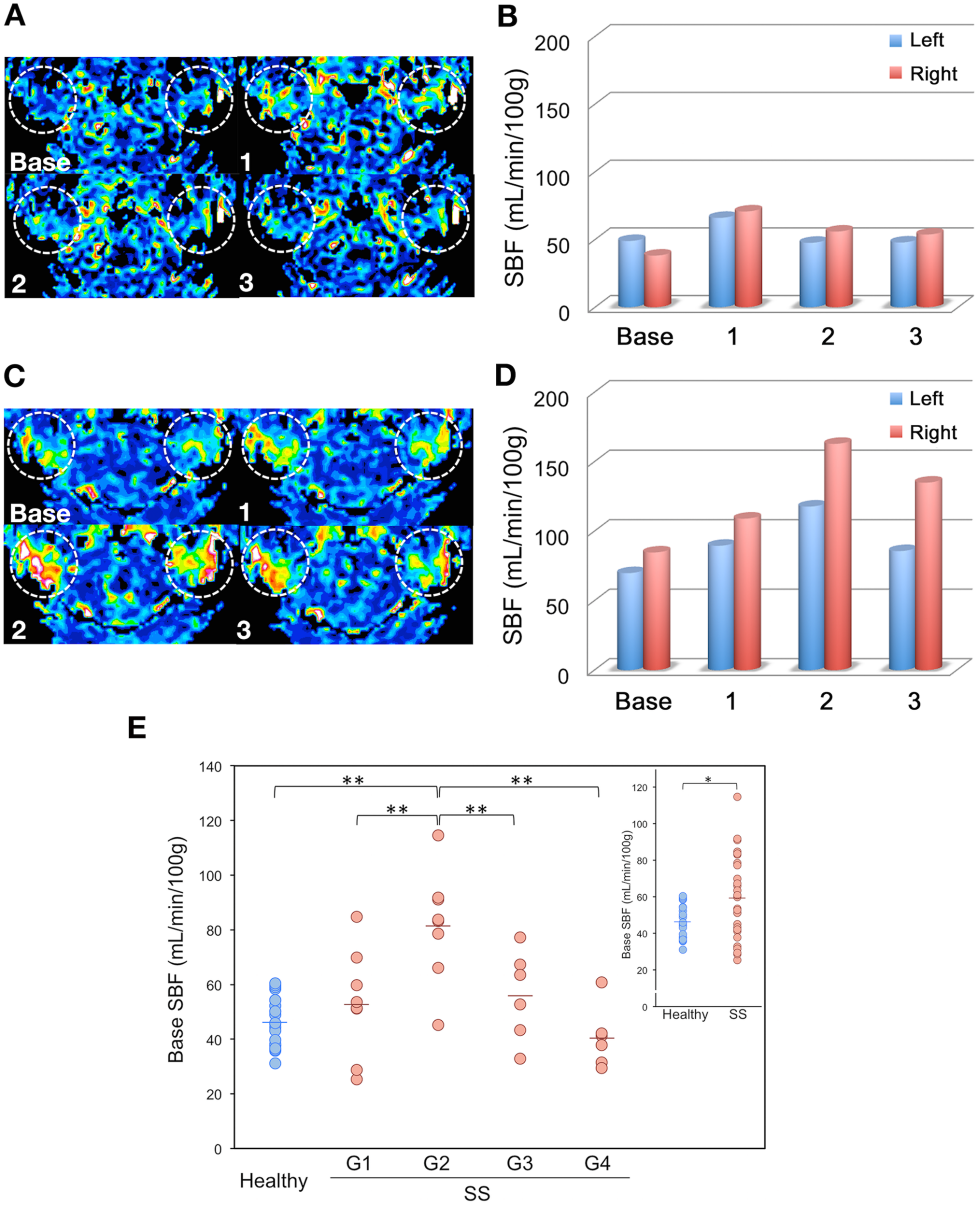
SBF kinetics of healthy and SS parotid glands. A, B, Axial color SBF maps (A) and bar graph for SBF levels (B) show Type 1 SBF kinetics profile from healthy parotid gland of 58-year-old woman (number 4 subject in Tables 1 and 2) before (Base) and after gustatory stimulation (Segments 1, 2, and 3). C, D, Axial color SBF maps (C) and bar graph for SBF levels (D) show Type 2 SBF kinetics profile from parotid gland of 48-year-old woman with SS (number 13 subject in Tables 1 and 2) before and after gustatory stimulation. E, Dot-plot graph shows base SBFs of 22 healthy and 28 SS parotid glands. *, significant difference for healthy vs. SS glands (p = 0.01, Welch test; inserted graph); **, significant differences for healthy vs. G2 SS glands (p <0.001), for G1 SS vs. G2 SS glands (p = 0.002), for G2 SS vs. G3 SS glands (p = 0.02), and for G2 SS vs. G4 SS glands (p < 0.001) (Tukey-Kramer HSD test).

The base SBFs of SS parotid glands were significantly higher than those of healthy glands (59.2 ± 22.8 vs. 46.3 ± 9.0 mL/min/100 mg, p = 0.01) (Fig 2A–2E, Table 2).

**Table 2:** SBF kinetic profiles of healthy and SS parotid glands.

SBF (mL/min/100 mg)															
Segment	Volunteer no. (left/right glands, average)														Total
	1	2	3	4	5	6	7	8	9	10	11				
Base	54.3/37.6	50.1/52.3	45.8/36.1	48.9/38.1	31.1/43.2	58.4/39.6	35.8/36.7	44.3/45.9	59.0/60.5	59.8/54.2	37.6/50.2				46.3 ± 9.0
	46.0	51.2	40.9	43.5	37.1	49.0	36.2	45.1	59.7	57.0	43.9				
1	**83.2/77.7**	**63.5/68.8**	**70.8/66.3**	**66.0/70.7**	**62.7/53.5**	**71.5/47.0**	**40.6/41.0**	**57.1/**51.6	**65.3/63.5**	**73.4/83.8**	**95.3/88.2**				66.4 ±14.4
	80.46	66.11	68.54	68.33	58.09	59.23	40.82	54.36	64.39	78.59	91.74				
2	42.2/37.5	43.0/56.3	67.7/53.8	47.5/55.8	45.9/48.6	65.1/43.1	41.0/38.6	56.4/53.0	63.3/58.0	61.7/73.5	57.8/73.3				53.8 ± 10.8
	39.81	49.67	60.71	51.69	47.24	54.12	39.82	54.65	60.64	67.63	65.53				
3	51.5/45.8	50.2/51.7	65.2/59.8	47.8/53.7	36.4/51.6	53.2/35.5	39.2/42.3	38.3/43.7	52.0/56.3	65.5/92.4	65.0/54.4				52.3 ± 12.6
	48.65	50.98	62.51	50.72	43.98	44.34	40.79	41.00	54.16	78.93	59.67				
Increment ratio	53/107	27/32	55/84	35/86	101/24	22/19	15/12	29/15	11/5	23/55	154/76				
SBF type	1/1	1/1	1/1	1/1	1/1	1/1	2/1	1/2	1/1	1/1	1/1				
Segment	SS patient no. (left/right glands, average)														Total
	12	13	14	15	16	17	18	19	20	21	22	23	24	25	
Base	51.2/51.3	69.9/84.7	25.4/28.7	59.8/53.5	83.1/114.6	78.5/45.1	91.0/66.1	83.7/91.7	77.2/52.7	43.3/32.8	67.4/63.5	41.2/31.5	37.9/29.4	42.1/60.8	59.2 ± 22.8
	51.3	77.3	27.0	56.7	98.9	61.8	78.6	87.7	65.0	38.1	65.5	36.4	33.6	51.5	
1	**63.7/71.2**	89.5/109.1	35.6/39.5	104.0/87.5	**98.3/193.0**	120.0/**99.4**	**104.0/73.3**	109.0/89.2	99.2/67.2	**56.1/43.7**	**83.8/**83.4	69.5/67.1	46.9/35.3	80.6/81.3	82.2 ± 32.1
	67.5	99.3	37.6	95.9	145.7	109.8	88.7	99.2	83.2	49.9	83.6	68.3	41.1	80.9	
2	56.7/62.7	**117.8/162.9**	**55.9/77.7**	**131.0/95.2**	83.8/156.0	**181.0/94.9**	103.0/63.3	**119.0/**102.2	**133.0/148.0**	39.5/31.9	65.6/**84.2**	**71.4/76.0**	**69.4/52.9**	**93.9/99.5**	93.8 ± 38.1
	59.7	140.4	66.8	113.0	119.9	137.8	83.3	110.4	140.3	35.7	74.9	73.7	61.1	96.7	
3	60.3/69.0	85.8/135	55.1/75.6	120/88.2	85.1/134	130/76.3	82.4/58.3	102/110.2	119/116	45.8/35.6	60.0/66.7	53.7/67.8	58.4/44.8	59.4/63.2	80.6 ± 29.1
	64.7	110.4	65.3	104.0	109.4	103.1	70.3	105.9	117.3	40.7	63.4	60.8	51.6	61.3	
increment ratio	24/39	69/92	120/171	119/78	18/68	130/120	14/11	42/20	72/181	29/33	24/33	73/141	83/80	123/64	
SBF type	1/1	2/2	2/2	2/2	1/1	2/1	1/1	2/3	2/2	1/1	1/2	2/2	2/2	2/2	

Bold SBF values indicate SBF peaks.

### SBP Peaks and Increment Ratios of Healthy and SS Parotid Glands

The SBF peaks of SS glands appeared in significantly later segments after gustatory stimulation than those of healthy glands (p < 0.001) (Fig 2A–2E, Tables 2 and 3). Accordingly, 20 (91%) of the 22 healthy glands were categorized into those with Type 1 SBF profiles with the SBF peak at the first segment and 2 (9%) had Type 2 SBF profiles with the SBF peak at the second segment; however, 17 (61%) of the 28 SS glands had Type 2 SBF profiles and only 10 (36%) of the glands exhibited Type 1 profiles (Tables 2 and 3). The Type 3 profile was noted in a single gland (4%). The SS glands showed significantly higher SBF increment ratios at peaks than did the healthy glands (p = 0.04) (Table 3).

**Table 3:** MR grades and SBF profiles of healthy and SS parotid glands.

	MR grades		SBF types			
		n	Type 1^a^	Type 2^a^	Type 3^a^	Increment ratio at SBF peak (%)^b^
Volunteers	G0	22	20	2	0	47 ± 39
SS patients	Total	28	10	17	1	74 ± 49
	G1	8	2	6	0	
	G2	8	5	2	1	
	G3	6	3	3	0	
	G4	6	0	6	0	

^**a**^, significant difference in SBF type distributions between healthy and SS glands (Fisher’s exact test, p <0.001).

^**b**^, significant difference in increment ratios at SBF peak between healthy and SS glands (p = 0.04, Mann-Whitney U test).

### Intra-Subject Variability of SBF Values

SBF values (base SBFs, SBF peaks, and increment ratios) often greatly differed between the left and right glands in the same individuals and SBF types of the left and right glands were different in some individuals (Table 2). Therefore, we assessed the intra-subject variability by calculating percent differences in SBF values between the left and right glands and then compared the percent differences in SBF values between different subject groups with different gland disease severity (G0—G4) and between subject groups with the same or different SBF types of left and right parotid glands in the same individuals. We found no correlations between the intra-subject variability in SBF values and the gland disease severity or the mismatch of SBF type (S1 Table).

### Correlations between SBF Profiles and Clinical Features of SS Patients

Base SBFs and SBF types had no significant correlations with the clinical features of SS patients (S2 Table). The healthy volunteers were significantly younger than the SS patients (Table 1). However, the age distributions in volunteers and SS patients were not significantly correlated with the distributions of SBF profiles (base SBFs and SBF types) in these 2 subject groups [p = 0.6090 for base SBFs (Pearson’s correlation coefficient test, correlation coefficient = 0.1075); and p = 0.4500 for SBF types (Spearman’s rank correlation test, rank correlation coefficient = 0.1582).

### SBF-based differentiation between healthy volunteers and SS patients

Lastly, we evaluated the base SBF as a diagnostic criterion for discriminating SS patients from healthy subjects. To this end, we performed ROC curve analysis of 2 different SBF criteria (average and maximal base SBF of the left and right parotid glands) for their diagnostic performance (Table 4). The analysis indicated that the average and maximal base SBFs provided equivalent diagnostic performance, albeit much lower sensitivity and higher specificity with the maximal SBF criterion.

**Table 4:** ROC curve analysis for the diagnostic abilities of base SBF values in differentiating between healthy volunteers and SS patients.

SBF criteria	SBF cutoff points (mL/min/100 mg)	diagnostic abilities (%)					AUC
		sensitivity	specificity	accuracy	PPV	NPV	
average base SBF	51.2	71	82	76	83	69	0.69
maximal base SBF	60.8	57	100	76	100	65	0.70

Diagnostic criteria are average or maximal salivary blood flow rates (SBFs) of left and right parotid glands. PPV and NPV, positive and negative predictive values. AUC, area under the receiver operating characteristic (ROC) curve.

## Discussion

In the present study, we compared the perfusion properties of SS parotid glands with those of healthy volunteers by using the 3T ASL technique before and after gustatory stimulation. We demonstrated that the SS glands are mostly hyperemic even in the resting states without gustatory stimulation and that the perfusion responses of the SS glands to the stimulation are stronger and longer-lasting than those of the healthy glands.

The elevated levels of base SBF in the parotid glands of patients with SS suggest a hyperemic state in the glands. These results are consistent with the Doppler waveform analysis data that the resistive and pulsatility indices without gustatory stimulation of the facial artery branch that supplies the submandibular gland were lower in patients with SS than in healthy volunteers [2]. More recently, dynamic contrast-enhanced MR imaging on the parotid glands showed that the levels of a fractional volume in the extravascular extracellular space, Ve, were markedly increased in SS glands compared to healthy glands, indirectly suggesting increased perfusion in the SS glands [11]. Minor salivary gland histologic features of patients with SS are defined as a focal lymphocytic sialadenitis, which are usually associated with non-specific chronic sialadenitis characterized by acinar atrophy, interstitial fibrosis and duct dilatation [12]. Although the predominant cell types that constitute the gland parenchyma are different between the parotid (serous acinar cells) and minor (mucous acinar cells) salivary glands, the fundamental pathologies may be similar [13]. Therefore, it is plausible to expect that chronic inflammatory changes similar to the minor salivary glands may occur in the parotid glands of patients with SS. These pathological changes in the SS glands may be associated with increased blood flow rates in the gland, which are consistent with the Doppler waveform and dynamic contrast-enhanced MR imaging results [2, 4, 11].

Recent studies have supported the notion that the endothelium-independent vasodilatation is impaired in the salivary glands of patients with SS [14, 15]. The decreased vascular responses in patients with primary SS suggested that the functional impairment of the endothelium is a result from disease-related chronic inflammation and immunologic factors [16]. In contrast to the previous studies, we found that the parotid glands of SS patients responded steadily to the gustatory stimulation. However, it should be noted that increased perfusion in resting states without gustatory stimulation was evident in the G2 glands, but not in the G1, G3, or G4 glands.

An alternative explanation for the delayed and prolonged responses of SS gland perfusion kinetics may be that the delay is caused by insufficient removal of the stimulant (i.e., lemon juice) from the surface of the tongue in SS patients whose salivary secretion is severely impaired. Inada et al. [17] reported that sour taste receptor PKD1L3-PKD2L1 ion channel complex can be activated only after the removal of the acid stimulus. Kamel et al. [18] reported that the taste threshold was reduced in patients with SS. Therefore, in patients with SS, the signaling pathway extending from the taste receptors to the acinar cells may be intact; however, chronic inflammation in the gland parenchyma impairs the function of endothelial cells, while the acinar cells are destroyed by infiltrating lymphocytes.

Initially, the ASL imaging technique was developed for assessing perfusion of the brain [6, 7]. Therefore, some factors used in the equation for determining SBF should be adjusted for the clinical assessment of salivary gland perfusion. In particular, the equation includes tissue T1 values specific for normal brain tissues. In the present study, we determined T1 values of the parotid glands in healthy volunteers and SS patients who were used for assessing SBF. The mean T1 value that was obtained from the parotid glands of the volunteers was slightly shorter than the authentic T1 value of the brain tissue (923 ms vs. 1200 ms), and the mean T1 value of the parotid glands of patients with SS was shorter (874 ms) than that of the healthy glands. The fat content in the parotid gland increases with age and in diseased states such as SS [10, 19]. However, a recent study showed that T1 values of the parotid glands did not change with increasing age [20]. Therefore, senile changes in the parotid glands may minimally affect the T1 value of the glands. However, pathological changes in the glands may significantly affect the T1 value. Indeed, the T1 values of the G4 glands were much shorter than those of the healthy and SS glands with the other disease grades. Therefore, SBF values determined with the ASL technique should be carefully interpreted when assessing glands with degenerative changes including SS glands.

Applications of ASL imaging to the extracranial tissues and organs have been very limited. Recently, Lanzman et al. [21] assessed the perfusion properties of renal tumors and found that ASL imaging effectively differentiated between some types of renal tumors based on the perfusion levels. These results imply that the ASL technique could help characterize salivary gland tumors with varying perfusion levels. Notably, effective differentiation between some types of benign and malignant tumors can be expected, considering previous findings that the perfusion-related parameter, D*, of Warthin tumor was significantly larger than that of malignant salivary gland tumors [22]. In fact, Kato et al. have recently shown that ASL imaging is useful for discriminating Warthin tumors from pleomorphic adenomas and malignant salivary gland tumors [23]. It may be of concern whether the T1 value of tumors should be determined for the calculation of tumor perfusion. Lanzman et al. [21] did not separately determine the T1value to calculate blood flow rates of renal masses because the tumor T1 values are generally long enough to minimize effects on the calculation of tumor perfusion. However, we found that fat tissue in the parotid glands, in particular in the glands of patients with SS, substantially shortened the T1 value. In the present study cohort, we found that SBFs increased by up to 20% compared to those calculated by using the authentic T1 value for the brain tissue (1200 ms) instead of using the T1 value for the parotid gland.

Application of ASL imaging for assessing the salivary gland function has been further limited. Of these, Schwenzer et al. [8] measured flow rates in healthy parotid glands. However, their results were much higher than those of the present study (335 mL/min/100 g at resting and 542 mL/min/100 g at peak), which were even higher than those reported for CBF (45–75 mL/min/100 g) [24]. On the other hand, our present results are consistent with those determined by CT perfusion study [25].

The functional assessment of the salivary glands can be performed by using diffusion-weighted (DW) imaging. Gustatory stimulation increases salivary flow rates, thereby increasing the water content of the salivary glands. Therefore, DW imaging can depict changes in the amount of saliva during gustatory stimulation. The water content in the glandular tissues can be estimated by measuring the apparent diffusion coefficient (ADC) of the gland. Thoeny et al. [26] investigated the ADC kinetics of the parotid gland after gustatory stimulation and found initial decreases in the ADC during the first 5–7 min after gustatory stimulation and latent increases during the following 15–20 min. They postulated that the initial decreases were attributable to the washout of stored saliva in the glands. It is of note that Kato et al. [27] observed initial increases, instead of decreases, in ADC after gustatory stimulation. Thoeny’s group used b-values of 400, 600, 800, and 1000 s/mm^2^, while Kato’s group used 0 and 1000 s/mm^2^. Therefore, the initial increases in the ADC reported by Kato et al. may have been affected by the rapid increases in perfusion within the glands in response to the gustatory stimulation, as observed in the present ASL study. These results suggest that the combined use of ASL and DW imaging could effectively characterize the impaired salivary gland function in patients with SS.

We found that tentative base SBF criteria could discriminate SS patients from healthy subjects of the present study cohort with moderate diagnostic efficacy. However, these results should be carefully interpreted considering that the controls did not have any dry mouth complaints and the differentiation based on the SBF values may be much ineffective in clinical situations, in which physician should discriminate SS patients from non-SS patients with sicca symptoms based on the AECG or ACR criteria. Therefore, a further study using a large cohort of patient with sicca symptoms is needed for evaluating the diagnostic ability of SBF parameters.

A major limitation of this study is that the T1 value for each gland was not available when calculating the SBFs. Determining the T1 values for each gland is time-consuming and cumbersome for routine clinical use. Therefore, innovations in determining tissue T1 value may be needed for the successful application of the ASL technique in assessing impaired salivary gland function. A recent study showed that the T1 values of the parotid glands did not change with increasing age [20]; however, pathological changes in the glands may significantly affect the T1 value. Indeed, the T1 values of the G4-stage glands were much shorter compared to those of both the healthy and G2 glands. Therefore, the SBF values determined using the ASL technique should be carefully interpreted when assessing glands with degenerative changes, including SS glands. The present study population was so small that the conclusive statement should await a future study using a larger patient population, which is also allowed with the innovation in T1 relaxation time measurement. In addition, 4 out of the 29 subjects were excluded form the study because of severe motion artifacts. The lack of consistency in obtaining adequate ASL images owing to motion artifacts may be a matter of considerable concern on the clinical application of the ASL technique.

### Conclusion

We conclude that the ASL MR imaging is applicable in assessing parotid gland perfusion and found that SS parotid glands were mostly hyperemic even in the resting states without gustatory stimulation and the gland perfusion responses to the stimulation were stronger and more prolonged than those of the healthy glands.

## Supporting Information

S1 Table Correlations between intra-subject variability in SBF values and gland disease grades. (DOCX)

S2 Table Correlations between the base SBFs/SBF types and the ages, salivary flow rates, disease durations, and parotid MR grades in SS patients. (DOCX)

S1 Text Image analysis and T1 relaxation time measurement. (DOCX)
