# Supplementary material for: Arterial Spin Labeling Imaging for the Parotid Glands of Patients with Sjögren’s Syndrome
Source: PLoS One. 2016 Mar 9;11(3):e0150680. doi: 10.1371/journal.pone.0150680 (PMC4784920; doi:10.1371/journal.pone.0150680)
Supplement: S1 Table — (DOCX) [file pone.0150680.s001.docx]

| **S1 Table. Correlations between intra-subject variability in SBF values and gland disease grades** | | | | | | | | |
| --- | --- | --- | --- | --- | --- | --- | --- | --- |
|  | intra-subject variability (percentage difference between left and right glands) | | | | | | | |
| SBF values | G0 (n =11) | G1 (n =4) | G2 (n = 4) | G3 (n = 3) | G4 (n = 3) | G1-4 (n = 14) | matched  SBF type | mismatched  SBF type |
| Base SBF  (mL/min/100 mg) | 20.3 ± 15.9^a^  [2.5 - 47.5] | 10.2 ± 6.3^a^  [0.2 - 17.5] | 37.0 - 23.8^a^  [8.7 - 74.1] | 28.2 ± 16.7^a^  [6.1 - 46.5] | 30.2 ± 0.88^a^  [28.9 - 30.8] | 26.0 ± 18.5^a^  [0.2 - 74.1] | 24.6 ± 13.9^b^  [0.2 - 47.5] | 19.0 ± 27.6^b^  [2.5 - 74.1] |
| SBF peak  (mL/min/100 mg) | 11.7 ± 13.5^a^  [0.2 - 52.1] | 25.9 ± 9.7^a^  [10.5 - 37.4 | 45.1 ± 26.3^a^  [7.6 - 81.7] | 13.1 ± 11.5^a^  [0.5 - 28.4] | 14.3 ± 12.0^a^  [5.6 - 31.2] | 26.2 ± 21.3^a^  [0.5 - 81.7] | 19.9 ± 15.4^c^  [2.8 - 52.1] | 19.5 ± 31.3^c^  [0.5 - 81.7] |
| Increment ratio | 81 ± 83^a^  [16 - 321] | 36 ± 10^a^  [25 - 53] | 55 ± 40^a^  [8 - 110] | 33 ± 20^a^  [12 - 60] | 48 ± 36^a^  [4 - 92] | 43 ± 30^a^  [4 - 110] | 62 ± 67^d^  [4 - 320] | 53 ± 41^d^  [8 - 110] |
| Data in square brackets indicate ranges of percentage difference in SBF value between left and right glands. Matched and mismatched SBF types, the same of different SBF types of left and right parotid glands in the same individuals.  a, Intra-subject variability is not significantly different in any combinations of subject groups (p >0.05; Steel-Dwass test).  b, c, d, Intra-subject variability is not significantly different (b, p = 0.709; c, p = 0.983; d, p = 0.725; Mann-Whitney U-test). | | | | | | | | |
