# Supplementary material for: Arterial Spin Labeling Imaging for the Parotid Glands of Patients with Sjögren’s Syndrome
Source: PLoS One. 2016 Mar 9;11(3):e0150680. doi: 10.1371/journal.pone.0150680 (PMC4784920; doi:10.1371/journal.pone.0150680)
Supplement: S2 Table — (DOCX) [file pone.0150680.s002.docx]

| **S2 Table. Correlations between the base SBFs/SBF types and the ages, salivary flow rates, diseases durations, and parotid MR grades in SS patients** | | | | |
| --- | --- | --- | --- | --- |
|  | p values (correlation coefficients) | | | |
|  | age | salivary flow rate | disease duration | MR grade |
|  |  |  |  |  |
| Base SBF | -0.0965 (0.7427)^a^ | 0.3011 (0.2955)^b^ | 0.7253 (0.1033)^b^ | -0.2223 (0.4449)^b^ |
| SBF type | -0.3252 (0.2565)^b^ | 0.1547 (0.5975)^b^ | 0.8021 (-0.0738)^b^ | 0.1130 (0.7005)^b^ |
|  |  |  |  |  |
| An average of left and right parotid gland base SBFs and a sum score of left and right gland numerical type (0-3) were assigned to each SS patient as an individual base SBF and SBF type, respectively, and then correlations between the individual SBF profiles (base SBFs and SBF types) and the clinical features [ages, salivary flow rates (g/2 min), disease durations (in month) and MR grades (1-4)] were assessed by using (a) Pearson’ s correlation coefficient test or (b) Spearman’s rank correlation coefficient test. A p value of <0.05 was considered statistically significant. | | | | |
