# Supplementary material for: Arterial Spin Labeling Imaging for the Parotid Glands of Patients with Sjögren’s Syndrome
Source: PLoS One. 2016 Mar 9;11(3):e0150680. doi: 10.1371/journal.pone.0150680 (PMC4784920; doi:10.1371/journal.pone.0150680)
Supplement: S1 Text — (DOCX) [file pone.0150680.s003.docx]

S1 Text

Image analysis

The original algorithm that is originally equipped for estimating the cerebral blood flow (CBF) is:

$CBF=6000\cdot\lambda\frac{\left[ 1- e^{-\frac{\mathrm{ST}}{T_{1t}}} \right]e^{\frac{\mathrm{PLD}}{T_{1b}}}}{2\varepsilon T_{1b}\left[ 1- e^{-\frac{\mathrm{LT}}{T_{1b}}} \right]\mathrm{NEX}_{\mathrm{PW}}} \left[ \frac{\mathrm{PW}}{SF_{\mathrm{PW}}\mathrm{PD}} \right]$ (1)

where the T1 value of blood (T_1b_) = 1.6 s at 3.0T; the partition coefficient (λ) = 0.9; the labeling efficiency (ε) = 0.6; the labeling duration (LT) = 1.5 s; the postlabeling delay (PLD) = 1525 ms; and the saturation time (ST) = 2 s; PD is the proton density-weighted image as the reference image; PW is the perfusion-weighted or the raw difference image; and SF_PW_ is the scaling factor of PW sequence; and NEXpw is the number of excitations for the PW images. In the original equation, T1 value is assumed to be 1.2 s for the brain.

However, this equation contains a predefined parameter of brain tissue T1 value (T_1t_). Therefore, the salivary blood flow (SBF) was estimated by multiplying the obtained CBF values by a factor that was calculated by the following equation:

$\frac{1-e^{-\frac{\mathrm{ST}}{T1p}}}{1-e^{-\frac{\mathrm{ST}}{T1t}}}$ (2)where the T_1p_ is T1 value of the parotid gland.

T1 relaxation time measurement

T1 relaxation time measurement required extra image acquisition times (40 min and 36 sec) in addition to the ASL imaging. The prolonged imaging time lead to unexpected head movements in many of the subjects with resultant errors in T1 value calculation, and some of them refused the repeated T1 relaxation time measurements. The T1 values were measured by using an inversion-recovery spin-echo imaging sequence with a fixed TE of 10 ms and TR of 7000 ms. T1 values were calculated by fitting the signal as a nonlinear least square method by using varying inversion time (TI) values of 50, 100, 800, 1200, 2000, and 4000 ms based on a three-parameter model as shown in the following equation:

$S= S_{0} \left[ 1-C\cdot e^{-\frac{\mathrm{TI}}{T_{1}}} \right]$ (3)

where S is the signal intensity at a particular inversion time, S_0_ is the equilibrium signal, and C is a parameter related to inversion efficiency and recovery time [8]. We used averaged T1 value of 923.0 ms for the healthy glands in calculating SBFs. The parotid glands contain increasing amounts of fat tissues in the gland parenchyma of SS patients with higher severity of gland disease. Therefore, we used separate averaged T1 values for the glands with different disease severity; 1026 ms for the G1, 938.2 ms for the G2, 814.7 ms for the G3, and 588.6 ms for the G4 gland.
